# Bioinformatic analysis of the molecular mechanism underlying bronchial pulmonary dysplasia using a text mining approach

**DOI:** 10.1097/MD.0000000000018493

**Published:** 2019-12-27

**Authors:** Weitao Zhou, Fei Shao, Jing Li

**Affiliations:** a Department of Pediatrics, The First Affiliated Hospital of the University of Science and Technology of China; b Department of Oncology, Second Affiliated Hospital of Anhui Medical University, Hefei; c Department of Pediatric Intensive Care Unit, Children's Hospital of Chongqing Medical University; Ministry of Education Key Laboratory of Child Development and Disorders; National Clinical Research Center for Child Health and Disorders; China International Science and Technology Cooperation base of Child Development and Critical Disorders; Children's Hospital of Chongqing Medical University; d Chongqing Key Laboratory of Pediatrics, Chongqing, China.

**Keywords:** bronchial pulmonary dysplasia, PI3K-AKT signaling pathway, systematic analysis, text mining

## Abstract

Bronchopulmonary dysplasia (BPD) is a common disease of premature infants with very low birth weight. The mechanism is inconclusive. The aim of this study is to systematically explore BPD-related genes and characterize their functions.

Natural language processing analysis was used to identify BPD-related genes. Gene data were extracted from PubMed database. Gene ontology, pathway, and network analysis were carried out, and the result was integrated with corresponding database.

In this study, 216 genes were identified as BPD-related genes with *P* < .05, and 30 pathways were identified as significant. A network of BPD-related genes was also constructed with 17 hub genes identified. In particular, phosphatidyl inositol-3-enzyme-serine/threonine kinase signaling pathway involved the largest number of genes. Insulin was found to be a promising candidate gene related with BPD, suggesting that it may serve as an effective therapeutic target.

Our data may help to better understand the molecular mechanisms underlying BPD. However, the mechanisms of BPD are elusive, and further studies are needed.

## 1 Introduction

Bronchial pulmonary dysplasia (bronchopulmonary dysplasia [BPD]) is a common disease of premature infants with low birth weight, characterized by continuing oxygen therapy at least 28 days, accompanied by pulmonary imaging changes.^[1]^ Pulmonary surfactant and small tidal volume aeration can alleviate lung injury and cut down premature infant mortality, but not decrease the incident rate of BPD. Recent studies show that 1 in every 10,000 children in the United States is diagnosed with BPD.^[2]^ The etiology of BPD is mainly due to the genetic susceptibility, oxygen toxicity, air pressure injury, capacity injury, infection, immunity and so on, resulting in oxygen toxicity and pulmonary fibrosis. However, the pathogenic factor is not clear.^[3]^ With the development of technique in molecular biology and genetic engineering, studies on molecular mechanism underlying the pathogenesis of BPD have been increasing in recent decades. The pathogenesis of BPD has been shown to be resulted from genetic susceptibility, infection, immature lung development, high concentration of oxygen injury, nutritional deficiency, and other factors.^[4]^

A large number of literatures show that the pathogenesis of BPD is attributed to some important protein and signaling pathways, such as angiotensin-converting enzyme, mannose-binding lectin, nuclear factor kappa B, phosphatidyl inositol-3-enzyme-serine/threonine kinase (PI3K-AKT) signaling pathway, interleukin (IL), vascular endothelial growth factor (VEGF).[^5^,^6^,^7^,^8^] Although a large number of transcription factors or signaling proteins have been discovered, studies on these transcription factors tend to be more conservative. Furthermore, defining the most meaningful regulatory factors from the information network is an important project.^[9]^

Due to the development of high-throughput proteomic and transcriptomic approach, it is feasible to study tens of thousands of genes and proteins at the same time nowadays. However, the results of high-throughput data are often lack of consistency due to the different choices of data platform and statistical criteria.^[10]^ Moreover, most valuable information remains hidden within literatures through conventional gene-by-gene way. In recent years, text mining has been used as an effective method to explore underlying mechanisms for many diseases.[^11^,^12^] It provides a way to retrieve data from published researches automatically.^[13]^ Text mining, as an effective method for the study of molecular mechanism, has been utilized in many diseases,^[14]^ such as: glioblastoma,^[15]^ endometrial cancer,^[16]^ breast cancer,[^17^,^18^] ectopic pregnancy,^[19]^ decidualization.^[20]^

In an attempt to provide a better understanding of BPD and pave a foundation for the development of novel therapeutic interventions in BPD, we performed a text mining analysis to identify genes related to BPD. The filtered gene set was subsequently carried out to systematically analyze their functions, and define signaling pathways and network involved.

## 2 Methods

### 2.1 Natural Language processing (NLP) analysis of BPD

**Figure 1:**
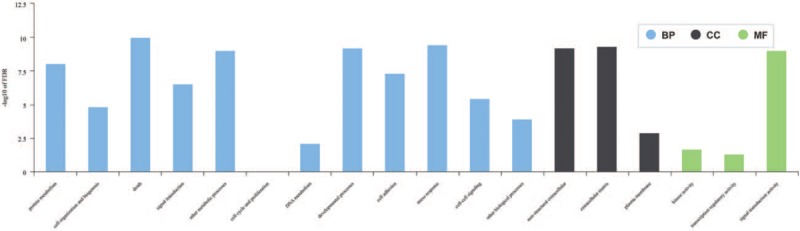
The histogram of Go terms enriched among bronchopulmonary dysplasia candidate genes according to BP, CC, and MF. BP = biological process, CC = cellular component, MF = molecular function.

PubMed database was used as the source of literature for text mining. The search was carefully conducted with the query term “bronchopulmonary dysplasia.” All relevant articles identified till March 2018 were retrieved and converted into XML format. All the genes and proteins associated with the query term were dug out and added to the list, followed by Gene mention tagging using a biomedical named entity recognizer software.^[21]^ Conjunction resolution was also carried out to identify individual descriptions on the extracted genes. In this study, gene symbol in entrez gene database of the National Center for Biotechnology Information was commonly used.[^22^,^23^] The processing flow chart of the NLP analysis of bronchopulmonary-dysplasia was shown in Figure 1.

For each gene, the frequency of its occurrence was denoted. The higher frequency a gene has, the higher correlation between the certain gene and BPD. The total number of publications retrieved from the PubMed database was recorded as *N*. The frequency of the certain gene and BPD were denoted by *m* and *n*, respectively.

*K* was used to represent the occurrence of both the gene and BPD in actual situations. Then, we calculated the probability of the frequency greater than k simultaneously cited under the completely random conditions by using hypergeometric distribution: 

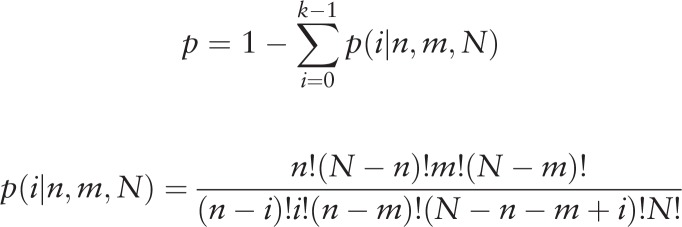



The BPD-gene relations with *P*-value < .05 were retrieved for further use.

### 2.2 Gene ontology (GO) analysis

GO analysis was performed by using the GSEABase package from R (http://www.r-project.org/) statistical platform. Word cloud was generated by using the word cloud package from R.^[24]^ The biological process (BP), cellular component (CC), and molecular function (MF) were characterized and evaluated in this study.

### 2.3 Pathway analysis

The Kyoto Encyclopedia of Genes and Genomes (KEGG) database was used to analyze the interactions between genes. Genes were mapped to the database by using GenMAPP v2.1 (http://www.genmapp.org/). Then statistical tests were performed to identify enriched pathways. *P* < .05 was set as threshold.^[25]^

### 2.4 Network analysis of BPD genes

We integrated the relationships of involved genes into 3 different interaction categories:

(1) protein interaction, gene regulation, protein modification listed in the KEGG database;
(2) interaction data from existing high-throughput protein interaction experiments, such as protein-protein interactions (PPrel) confirmed by yeast 2-hybrid;
(3) gene interaction demonstrated in previous publications.

Briefly, the pathway data were downloaded from the KEGG database, and then used to analyze the interaction among genes. We also downloaded KEGGSOAP package from R statistical analysis platform (http://www.bioconductor.org/packages/2.4/bioc/html/KEGGSOAP.html). Three different types of relationships were analyzed: enzyme-enzyme relation (ECrel, indicating 2 enzymes catalyzing successive reaction steps), PPrel (such as binding and modification), gene expression interaction (GErel, indicating relation of transcription factors and target gene products).^[26]^ The PPrel data was obtained by using the mammalian protein-protein interaction database (http://mips.helmholtz-muenchen.de/proj/ppi).^[27]^ For interactions that had already been published, we used co-citation matrices in PubMed. Using this algorithm, we identified the certain gene terms and co-occurred term variants within the sentences of an abstract. The frequency of the co-cited genes was analyzed as well. Finally, statistical analysis was performed using the hypergeometric distribution as described above. The resulting network of relationship was built and displayed by Medusa software.^[28]^ Then, the genes with a large number of connections that play important roles in the network stability were identified.

## 3 Results

### 3.1 NLP analysis of BPD

After the retrieval of documents from Pubmed, a total number of 5812 primary studies were identified, and 397 were obtained. Eventually, 216 genes were identified as BPD-related genes with *P* < .05. Among these genes, vascular endothelial growth factor A (VEGFA), IL-6, tumor necrosis factor (TNF), and interleukin 1, beta were mentioned most frequently. The top 20 frequently cited genes were listed in Table 1

**Table 1:**
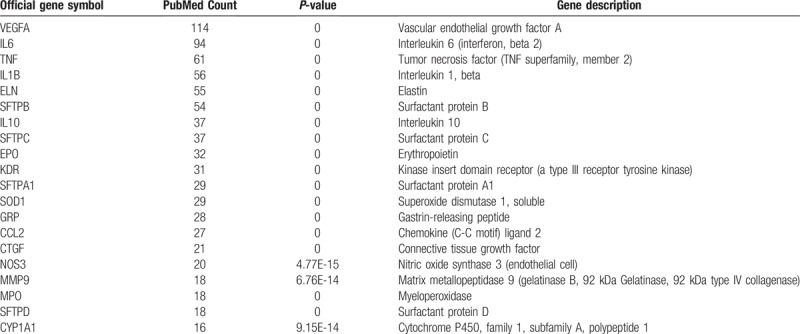
The top 20 frequently cited genes related to bronchopulmonary dysplasia.

### 3.2 GO analysis

These 216 genes mentioned above were categorized in GO analysis. Enriched GO terms are classified according to BP, CC, and MF. In the BP category, 12 GO terms, namely protein metabolism, cell organization and biogenesis, death, signal transduction, other metabolic processes, cell cycle and proliferation, DNA metabolism, developmental processes, cell adhesion, stress response, cell-cell signaling, and other BP were found to be significantly enriched. GO terms related to nonstructural extracellular, extracellular matrix, and plasma membrane region were significantly enriched under the CC category. Enriched GO terms in the MF category included kinase activity, transcription regulatory activity, signal transduction activity (Fig. 1).

### 3.3 Pathway analysis and gene network

**Figure 2:**
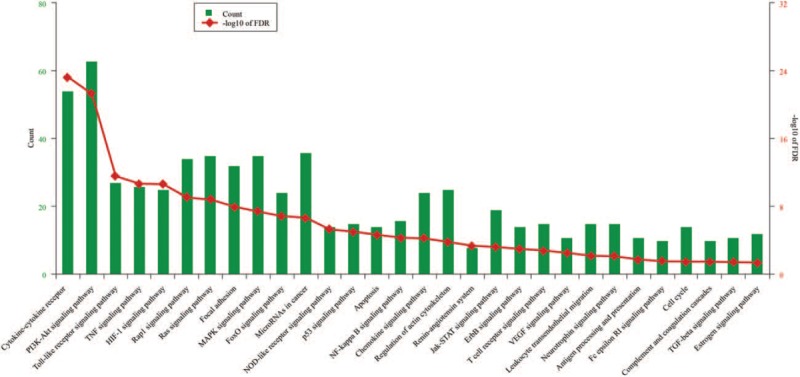
The significantly enriched pathways identified by using DAVID online tools.

**Figure 3:**
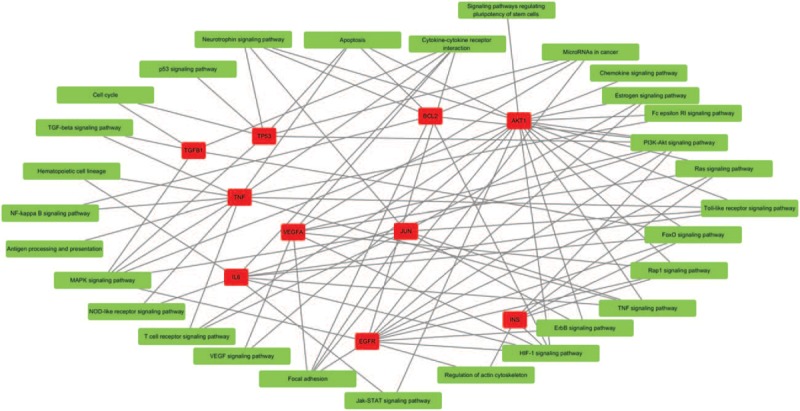
Pathway assignment for the top 10 genes recommended by the TM-rank algorithm. This graph was made by Cytoscape software. Ellipse nodes in red represent genes and rectangle nodes in blue represent pathways.

To better understand the gene function related to BPD, we also performed pathway analysis by using DAVID online tools. All BPD-related genes were mapped to the pathways found in KEGG. As shown in Figure 2, 30 signaling pathways were identified as significant. Pathway assignment for the top 10 genes was recommended by the TM-rank algorithm and generated by Cytoscape software (Fig. 3).

**Figure 4:**
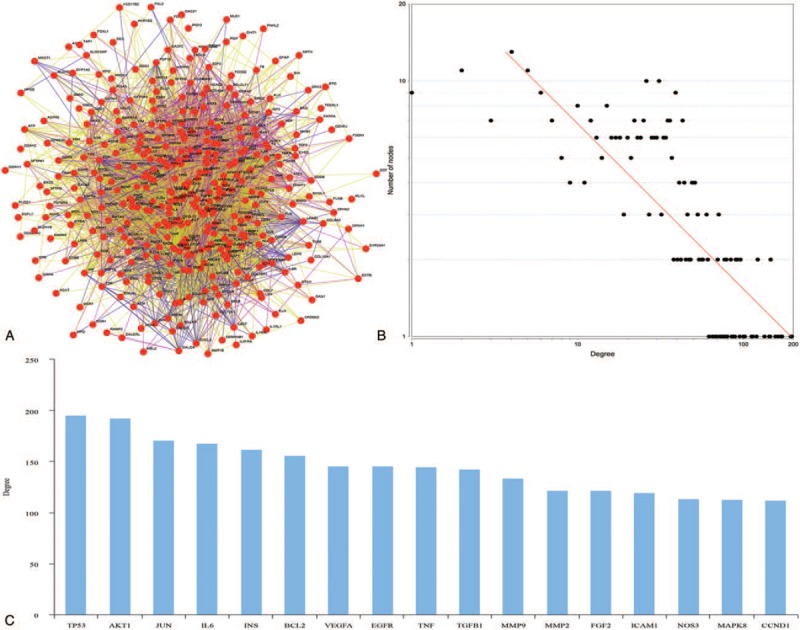
The gene network underlying all bronchopulmonary dysplasia candidate genes. (A) The structure of the gene network generated by using the MIPS database. (B) Degree distribution of the gene network. (C) The seventeen hub genes identified in the network. MIPS = mammalian protein-protein interaction.

We constructed a network of BPD-related genes that consists of 365 nodes connected via 6403 edges (Fig. 4A). Topological analysis proved that the network followed a power-law distribution (Fig. 4B). In this network, we identified 17 hub genes (Fig. 4C): tumor protein (TP53), v-akt murine thymoma viral oncogene homolog 1, jun oncogene, IL6, insulin (INS), B-cell CLL/lymphoma 2, VEGFA, epidermal growth factor receptor, TNF, transforming growth factor, beta 1, matrix metallopeptidase 9, matrix metallopeptidase 2, fibroblast growth factor 2, intercellular adhesion molecule 1, nitric oxide synthase 3, mitogen-activated protein kinase 8, and cyclin D1. Among these genes, TP53 was the BPD-related gene that exhibited the greatest number of interactions.

## 4 Discussion

A total number of 216 BPD-related genes were identified from the literature search of 5812 publications. Among the BPD-related genes we identified in the search, VEGFA, IL6 were the most frequently mentioned. Studies have already shown that there is a relevant association between BPD development and VEGFA activity in preterm infants.[^29^,^30^] VFGFA was shown to play vital roles in the repair of vascular lung, and the lack of VEGFA activity could lead to an impairing lung microvascular development in fetus.^[31]^ IL6 expression was found to be increased with the histologic severity of chorioamnionitis as well.^[32]^

The role of BPD-related genes could be validated through wet experiments theoretically; however, due to the large number of genes involved, experimental work is not a feasible for us to get a comprehensive understanding of the gene set. Thus, we performed pathway enrichment analysis using the complete list of 216 candidate genes by using the DAVID tools. A total of 30 enriched pathways were identified. Of particular interest was the PI3K-Akt signaling pathway. Notably, 67 BPD-related genes were enriched in this pathway. PI3K becomes activated by INS and is responsible for most of the metabolic actions mediated by INS. PI3K plays essential roles in many metabolic process such as cell growth, differentiation, survival, and protein synthesis.^[33]^ Inhibition of the PI3K-Akt pathway disrupts normal lung development, whereas the activation of PI3K-Akt pathway preserves alveolar development.^[34]^ PI3K-Akt signaling pathway could exert a cytoprotective role in cell survival during hyperoxia.^[35]^

We interpret that text mining result is usually based on the frequency calculation for each gene in publications. However, the most popular genes are not always the most important ones. It is known that, rather than working alone, gene products such as proteins usually form complexes to exert their functions.^[36]^ Hence, the functional importance of a gene depends on its interaction with other partners. The hub genes are likely more important due to their key positions in the ECrel, PPrel and GErel network. In this study, we constructed a large network of BPD-related genes that consists of 365 nodes connected via 6403 edges. Seventeen hub genes were identified in this network. Even the noisiness and incompleteness of interaction data may cause the inaccuracy of our results, the network could still provide us a comprehensive and reasonable way to understand the gene set.

Text Mining is characterized as the way toward separating meaningful information from numerous unstructured text utilizing computational methods. However, these articles of high- throughput experiments generate a large amount of gene information sometimes cannot be fully recognized and refined. For these articles, we downloaded full texts (as well as supplementary files if needed) and extracted gene mentions by hands. To reduce false-positive results, full texts of extracted papers contained hub-gene were downloaded, followed by manual confirmation of information. For most of the hub-genes, their relationships with BPD were easily understood and well-studied. However, when it comes to INS, most extracted papers are actually about insulin-like growth factor-I instead of INS. Only 2 true positive studies were found. One study mentioned that the serum INS is significantly increased in BPD infants after the administration of systemic corticosteroid treatment.^[37]^ Postnatal application of glucocorticoids can prevent BPD in preterm infants. However, the treatment also has adverse effects such as hyperglycemia, hypertension, and intestinal perforation. Serum INS level is elevated as a result of hyperglycemia.^[38]^ However, the role of INS in BPD is not clear. The other found that INS could increase cell function and NO production in normal fetal pulmonary artery endothelial cells (PAECs). PAECs from intrauterine growth restriction fetuses were less sensitive to INS which caused significantly decreased cell motility, growth, tube formation, and NO production. Impaired PAEC function may contribute to the increased rate of BPD.^[39]^ Furthermore, a related paper on Metformin reversing established lung fibrosis in a bleomycin model was published in 2008; it implicated INS in alleviating the later pathological change of BPD.^[40]^ Usually, the limitation for text mining–based strategies is that there is no chance to discover new genes.^[19]^ Interestingly, in this study we found INS as a novel gene related to BPD by accident. This could pave a foundation for further investigation about the molecular mechanisms between INS and BPD. As a matter of fact, our purpose of this work is to provide a foundation for identifying new molecules that are worthy perusing in the future, rather than summarizing previous works. Genes not in the central position of the network or do not have direct interactions with principal molecules could still be land for novel findings.

In conclusion, we systematically analyzed BPD-related genes using a text mining approach. These genes were further characterized by GO, pathway and network analysis. Our research provides a basis for a better understanding of the molecular mechanisms underlying BPD and paves a foundation for further studies as well.

## Acknowledgment

These authors wish to express their gratitude to the Shanghai Boyun Biotechnology Co. Ltd, for bioinformatics analysis.

## Author contributions

**Conceptualization:** Fei Shao, Jing Li.

**Data curation:** Weitao Zhou, Fei Shao.

**Formal analysis:** Weitao Zhou, Fei Shao.

**Funding acquisition:** Jing Li.

**Methodology:** Weitao Zhou, Fei Shao.

**Supervision:** Jing Li.

**Writing – original draft:** Weitao Zhou, Fei Shao.

**Writing – review and editing:** Weitao Zhou, Fei Shao.

Weitao Zhou orcid: 0000-0003-3618-5193.
